# Serovars and Antimicrobial Resistance of *Salmonella* in Food Workers and Livestock Products: Insights into Foodborne Transmission Pathways in Eastern Japan

**DOI:** 10.3390/pathogens14100958

**Published:** 2025-09-23

**Authors:** Yoshimasa Sasaki, Kenji Ohya, Yoshika Momose, Masashi Uema, Tetsuya Ikeda, Mizuki Sasaki, Tetsuo Asai

**Affiliations:** 1 Division of Veterinary Science, Department of Veterinary Medicine, Obihiro University of Agriculture and Veterinary Medicine, Inada-cho, Obihiro 080-8555, Hokkaido, Japan; msasaki@obihiro.ac.jp; 2 Division of Microbiology, National Institute of Health Sciences, 3-25-26, Tonomachi, Kawasaki-ku, Kawasaki 210-9501, Kanagawa, Japan; 3 Division of Biomedical Food Research, National Institute of Health Sciences, 3-25-26, Tonomachi, Kawasaki-ku, Kawasaki 210-9501, Kanagawa, Japan; momo@nihs.go.jp (Y.M.); m-uema@nihs.go.jp (M.U.); 4 Department of Infectious Diseases, Hokkaido Institute of Public Health, Kita19 Nishi 12, Kita-ku, Sapporo 060-0819, Hokkaido, Japan; ikeda@iph.pref.hokkaido.jp; 5 Department of Applied Veterinary Science, The United Graduate School of Veterinary Science, Gifu University, 1-1, Yanagido, Gifu 501-1193, Japan; asai.tetsuo.x3@f.gifu-u.ac.jp

**Keywords:** antimicrobial resistance, food worker, livestock product, *Salmonella*

## Abstract

*Salmonella* is a major cause of infectious enteritis worldwide. In Japan, *S*. Schwarzengrund, *S*. Infantis, and *S*. Thompson are common in broilers and laying hens and are frequently detected in patients with salmonellosis and food workers. Monophasic *S*. Typhimurium, also found in these populations, often exhibits multidrug resistance. However, multidrug-resistant monophasic *S*. Typhimurium has not been reported from domestic poultry, suggesting that other livestock products may be potential sources. Therefore, we examined *Salmonella* prevalence in retail pork, beef, and quail eggs, and characterized isolates from these products and from food workers using serotyping, antimicrobial susceptibility testing, and multilocus sequence typing. *Salmonella* was highly prevalent in pork liver (43.3%, 13/30) and imported chicken (20.7%, 18/87). Eleven pork liver isolates and two imported chicken isolates (Brazil and Thailand) were multidrug-resistant monophasic *S*. Typhimurium sequence type (ST) 34. Among 232 isolates from food workers, monophasic *S*. Typhimurium was the third most frequent serovar, with 63.2% (12/19) being multidrug-resistant ST34. *Salmonella* was not detected in beef. Hence, food workers may acquire multidrug-resistant monophasic *S*. Typhimurium ST34 through contaminated pork liver and imported chicken. Thorough cooking of chicken and pork meat, including liver, is essential to reduce the risk of *Salmonella* transmission.

## 1. Introduction

Non-typhoidal *Salmonella* is an important zoonotic pathogenic agent, causing an estimated 93.8 million cases of gastroenteritis and 155,000 deaths worldwide annually [1]. The World Health Organization has identified non-typhoidal *Salmonella* as one of the four key global causative agents of diarrhea, noting that salmonellosis in humans is generally contracted through the consumption of contaminated food of animal origin (mainly eggs, meat, and milk) [2]. In accordance with the Japanese Food Sanitation Act, physicians are required to report food poisoning diagnoses to the local public health center. The incidence of *Salmonella*-related foodborne outbreaks in Japan decreased from 262 in 2000 to 15 in 2018. Nevertheless, these outbreaks still rank as the second most common bacterial food poisoning outbreak in the country, and the number of patients per outbreak has shown an increasing trend [3]. Identifying the important sources and transmission routes of foodborne pathogens is crucial for developing food safety strategies. However, identifying the food sources in most cases of *Salmonella*-related foodborne outbreaks is challenging. Critical reasons are that by the time the incident is reported to a public health center by a physician, the implicated food may no longer be available for analysis, the patient may have ingested multiple food items during the incubation period, or the patient may not accurately recall what they consumed.

In Japan, large-scale food service companies, including fast-food chains, central kitchens, and school lunch centers, regularly conduct stool tests for their food workers as part of their hygiene management protocols. The large number of such companies enables the continuous collection of *Salmonella* isolates by securing the cooperation of these companies, and the testing laboratories that conduct their stool examinations. This would provide a substantial advantage for epidemiological surveillance and research. As food workers not only consume the food products manufactured at their workplaces but also eat a variety of other foods in their daily lives as ordinary consumers, monitoring the characteristics of *Salmonella* strains isolated from food workers could be useful for developing food safety strategies. In a previous study, we isolated *Salmonella* from 583 (0.079%) of 740,635 stool samples collected from food workers between January and December 2018, and the four most common serovars were *Salmonella* Schwarzengrund, *S*. Infantis, the monophasic *S*. Typhimurium, and *S*. Thompson [4]. *S*. Schwarzengrund and *S*. Infantis are the two most prevalent serovars isolated from local broilers in Japan [5], and *S*. Thompson and *S*. Infantis are among the top seven serovars isolated from domestic laying hens [6], whereas monophasic *S*. Typhimurium is rarely detected in broilers and laying hens [5,6,7]. Contrastingly, the monophasic *S*. Typhimurium is commonly isolated from local pigs and cattle in Japan [8,9]. Shimojima et al. [10] reported the isolation of the monophasic *S*. Typhimurium from imported chicken and pork. Moreover, outbreaks of food poisoning caused by the monophasic *S*. Typhimurium have been reported, and these outbreaks have been presumed to be linked to the consumption of raw quail eggs [11]. These findings suggest that the food workers may have been infected with the monophasic *S*. Typhimurium through the consumption of pork, beef, or quail eggs.

Therefore, we investigated the prevalence of *Salmonella* in pork, beef, and quail eggs, and characterized *Salmonella* isolated from these livestock products and food workers using serotyping, antimicrobial susceptibility testing, and multilocus sequence typing (MLST).

## 2. Materials and Methods

### 2.1. Sample Collection

The livestock products listed in Table 1 were purchased from 22 retail stores in Eastern Japan. The quail eggs were collected in boxes of 10 shell eggs. While the imported chicken thigh meat was frozen, all other food products were kept refrigerated at retail stores.

### 2.2. Salmonella Isolates Recovered from Food Workers Employed at a Single Fast-Food Company

Under anonymity, this study utilized 232 *Salmonella* isolates recovered from a total of 409,307 stool specimens collected and tested between January and December 2020 from food workers employed at outlets of a single fast-food chain in Eastern Japan. The food workers included cooks and servers in restaurants and food factory workers. The related stool tests were conducted regularly for food workers as part of the chain’s hygiene management protocol, and they were not performed specifically for this study. The adopted procedures for stool testing have been described in our previous report [4].

### 2.3. Salmonella Isolation from Livestock Products

The livestock products were transported under refrigeration to the National Institute of Health Sciences or the Obihiro University of Agriculture and Veterinary Medicine. Each sample was tested within 24 h after arrival at the laboratories. For meat and liver products, 25 g of the sample was mixed in 225 mL of buffered peptone water (BPW: Oxoid Ltd., Hampshire, UK; cat. no. CM0509) and incubated at 37 °C for 18 h for pre-enrichment. For quail egg samples, shell eggs were aseptically broken. Then, shell and egg contents from 10 raw shell eggs were separated and pooled in separate containers. For egg contents, whole egg contents from 10 eggs were placed in a plastic bag and mixed to homogeneity. Next, the egg contents were mixed with 400 mL of BPW and incubated at 37 °C for 18 h for pre-enrichment. The shells were crushed, mixed with 225 mL of BPW, and incubated at 37 °C for 18 h for pre-enrichment. After pre-enrichment, 0.1 and 1 mL of the culture were added to 10 mL of Rappaport–Vassiliadis broth (Oxoid Ltd., Hampshire, UK; cat. no. CM0669B) and 10 mL of Hajna tetrathionate broth (Eiken Chemical, Tokyo, Japan; cat. no. E-MA19), respectively, and the mixtures were then incubated at 42 °C for 20 h. After incubation, each culture was streaked onto two selective isolation agar plates: Xylose Lysine Deoxycholate agar (Oxoid Ltd., Hampshire, UK; cat. no. CM0469B) and CHROMagar^TM^ Salmonella (CHROMagar, Paris, France; cat. no. SA132), and the plates were then incubated at 37 °C for 24 h. Putative *Salmonella* colonies were biochemically identified [12]. One isolate per sample was suspended in 20% glycerol and stored at −80 °C until the mixture was ready for serotyping and antimicrobial susceptibility testing. For the monophasic *S*. Typhimurium, MLST was conducted. 

### 2.4. Serotyping

*Salmonella* isolates were tested for somatic antigens with slide agglutination using O antisera (Denka Co., Tokyo, Japan; cat. Nos. 200112 and 291875). *Salmonella* isolates were further tested for flagella antigens with tube agglutination using H antisera (Denka Co., Tokyo, Japan; cat. Nos 200129, 200136, 200143, 200150, and 200167). Serovars were determined based on the reaction between O and H group antigens according to the Kauffmann–White scheme [13]. Isolates agglutinated with anti-O4 and anti-H:i serum but not anti-H:1 or anti-H:2 serum were confirmed as the monophasic *S*. Typhimurium based on a previously reported polymerase chain reaction method [14].

### 2.5. Antimicrobial Susceptibility Testing

Minimum inhibitory concentrations of *Salmonella* isolates were determined with a two-fold broth microdilution method in 96-well microtiter plates (Dry-plate “Eiken,” Eiken Chemical, Tokyo, Japan), as described in the standards of the Clinical and Laboratory Standards Institute (CLSI) [15]. The following antimicrobial agents were tested: ampicillin (range of antimicrobial dilution: 1–128 mg/L), cefazolin (1–128 mg/L), cefotaxime (0.5–64 mg/L), streptomycin (1–128 mg/L), gentamicin (0.5–64 mg/L), kanamycin (1–128 mg/L), tetracycline (0.5–64 mg/L), nalidixic acid (1–128 mg/L), ciprofloxacin (0.03–4 mg/L), colistin (0.12–16 mg/L), chloramphenicol (1–128 mg/L), and trimethoprim (0.25–16 mg/L). The *Escherichia coli* reference strain ATCC 25922 was used as a control. The plates were incubated for 24 ± 2 h at 37 °C under aerobic conditions. The resistance breakpoints were defined based on the CLSI standard [16] and the Report on the Japanese Veterinary Antimicrobial Resistance Monitoring System 2016–2017 [17]. Multidrug resistance was defined as resistance to antimicrobial agents from two or more classes.

### 2.6. MLST

MLST of the monophasic *S*. Typhimurium was performed using nucleotide sequences of seven housekeeping genes (*aroC*, *dnaN*, *hemD*, *hisD*, *purE*, *sucA*, and *thrA*) according to protocols available on the MLST database (https://pubmlst.org/organisms/salmonella-spp/, accessed on 23 June 2025). 

### 2.7. Statistical Analysis

All statistical analyses were performed using R version 4.1. Differences between proportions were tested using Fisher’s exact test, and *p*-values < 0.05 were considered statistically significant.

## 3. Results

### 3.1. Salmonella Prevalence in Livestock Products

In quail eggs, *Salmonella* was isolated from one (1.7%) pooled eggshell sample but not from any of the pooled egg content samples (Table 1). In pork products, *Salmonella* was isolated from one (2.5%) minced meat sample and 13 (43.3%) liver samples, indicating a significantly (*p* < 0.05) higher prevalence in liver compared to minced meat. In beef products, *Salmonella* was not isolated from either minced meat or liver samples. In imported chicken products, *Salmonella* was isolated from 18 (20.7%) samples. *Salmonella* prevalence was significantly (*p* < 0.05) higher in samples produced in Thailand (60.0%) than in those produced in Brazil (13.2%). 

### 3.2. Serovars of Salmonella Isolates from Food Workers and Livestock Products

The isolates recovered from food workers were serotyped into 47 serovars and 7 untypeable *Salmonella* isolates (Table 2). The top six serovars (*S*. Schwarzengrund, *S*. Manhattan, monophasic *S*. Typhimurium, *S*. Thompson, *S*. Infantis, and *S*. Braenderup) collectively accounted for over 50.4% of isolates from food workers. In pork products, 12 (11 liver samples and 1 minced meat sample) and two isolates were the monophasic *S*. Typhimurium and *S*. Derby, respectively. In imported chicken products, the most common serovar was *S*. Minnesota (five isolates), and there were two isolates of the monophasic *S*. Typhimurium. The isolate recovered from one quail eggshell sample was the monophasic *S*. Typhimurium.

### 3.3. Antimicrobial Susceptibility of Salmonella Isolates and Sequence Types (STs) of the Monophasic S. typhimurium

The isolates from food workers exhibited high rates of antimicrobial resistance to streptomycin (48.7%) and tetracycline (29.7%) (Table 3). Two (0.9%) isolates (one *S*. Typhimurium isolate and one *S*. Anatum isolate) were resistant to cefotaxime. Three (1.3%) isolates (one *S*. Typhimurium isolate, one *S*. Kentucky isolate, and one untypeable isolate) were resistant to ciprofloxacin. The isolates from pork liver showed high rates of antimicrobial resistance to streptomycin (92.3%) and tetracycline (76.9%). The isolates from the imported chicken demonstrated high rates of antimicrobial resistance to ampicillin (66.7%) and tetracycline (61.1%). Six isolates (one *S*. Agona, one *S*. Heidelberg, and four *S*. Minnesota isolates) were resistant to cefotaxime. One *S*. Kentucky strain was resistant to ciprofloxacin. Nineteen isolates of the monophasic *S*. Typhimurium recovered from food workers were classified into two STs: ST19 (seven isolates) and ST34 (twelve isolates) (Table 4). Of the seven ST19 isolates, only one was multidrug-resistant. Conversely, all the ST34 isolates were multidrug-resistant. Moreover, all of the monophasic *S*. Typhimurium isolates recovered from pork products and imported chicken products belonged to ST34 and were multidrug-resistant. The monophasic *S*. Typhimurium isolate recovered from a quail eggshell sample belonged to ST19 and was susceptible to all of the antimicrobials tested in the study.

## 4. Discussion

The top five serovars in the *Salmonella* isolates from food workers were *S*. Schwarzengrund, *S*. Manhattan, monophasic *S*. Typhimurium, *S*. Thompson, and *S*. Infantis. Four of these serovars (all except *S*. Manhattan) were also among the top five serovars recovered from food workers in our previous work in 2018 [4]. Monophasic *S*. Typhimurium ranked third in both the present and 2018 studies, whereas *S*. Manhattan ranked sixth in the 2018 study. The antimicrobial resistance profiles of these serovars closely resembled those reported in 2018. For example, most *S*. Schwarzengrund isolates were resistant to streptomycin, kanamycin, and tetracycline, and most *S*. Manhattan isolates were resistant to streptomycin and tetracycline. Monophasic *S*. Typhimurium isolates frequently exhibited resistance to ampicillin, streptomycin, and tetracycline. Contrastingly, most *S*. Infantis isolates were susceptible to all tested antimicrobials. These findings suggest that the sources of human *Salmonella* infections remained mostly unchanged over the past 2 years. Supporting this, a previous report from Tokyo, Eastern Japan [18], indicated that the seven most common *Salmonella* serovars isolated between 2018 and 2020 from foodborne outbreaks, sporadic cases, and asymptomatic carriers were *S*. Schwarzengrund, *S*. Enteritidis, monophasic *S*. Typhimurium, *S*. Infantis, *S*. Typhimurium, *S*. Agona, and *S*. Thompson. Notably, these serovars overlap substantially with those identified in food workers in the present study. Together, these findings suggest that routine monitoring of *Salmonella* isolates from the stool of food workers may enable temporal estimation of serovars potentially responsible for human salmonellosis and identification of their likely food sources. Nevertheless, isolates recovered from stool samples are not necessarily pathogenic to humans. 

Yoshikura et al. [3] analyzed food poisoning statistics published by the Ministry of Health, Labour and Welfare of Japan and found that the main sources of *Salmonella* food poisoning were chicken meat and chicken eggs. *S*. Schwarszengrund, *S*. Manhattan, and *S*. Infantis are among the frequently isolated serovars from broilers in Japan [5]. *S*. Thompson and *S*. Infantis are among the frequently isolated serovars from laying hens in Japan [6]. Food workers and patients with salmonellosis are presumed to contract *Salmonella* infection through the consumption of chicken meat and eggs. Monophasic *S*. Typhimurium is frequently isolated from domestic cattle and pigs in Japan [9,17]. Therefore, it was expected to be commonly isolated from beef and pork products. However, it was not isolated from any of the beef products tested. Contrastingly, it was isolated in one minced pork meat sample (2.5%) and at a high rate (36.7%; 11/30) in pork liver samples. In a survey conducted at slaughterhouses in Germany [19], *Salmonella* was isolated from 0.3% (3/865) of pork cuts and 4.5% (5/110) of pork livers, whereas no *Salmonella* was isolated from beef cuts (283 samples) or livers (66 samples). A study reported that *Salmonella* was not isolated from 44 samples of ground beef and 44 samples of beef liver collected from retail stores in Nashville, Tennessee, USA [20]. Collectively, these findings indicate that the prevalence of *Salmonella* in beef meat and liver is considerably lower than that observed in pork products. Additionally, *Salmonella* was isolated from two imported chicken and one quail eggshell samples. A study conducted in Brazil isolated multidrug-resistant monophasic *S*. Typhimurium ST34 from chilled chicken meat samples collected from retail markets [21]. The multidrug-resistant monophasic *S*. Typhimurium isolates obtained from food workers were classified as ST34, except for one isolate that belonged to ST19. All monophasic *S*. Typhimurium isolates recovered from pork and imported chicken were multidrug-resistant and classified as ST34. To our knowledge, there are no reports from Japan of multidrug-resistant monophasic *S*. Typhimurium being isolated from domestic poultry, broilers, or laying hens [5,6,7,17]. Based on this, food workers may have been infected with multidrug-resistant monophasic *S*. Typhimurium via the consumption of pork and imported chicken products. However, to elucidate the infection routes of monophasic *S*. Typhimurium belonging to ST19 among food workers, it is necessary to investigate a wider variety of food products and food-producing animals. Arai et al. [22] characterized the genotypes of monophasic *S*. Typhimurium isolated from food-producing animals and found that ST19 was present until the early 2010s. Since then, ST34, which spread from Europe, has become increasingly prevalent. We identified the genotypes of 13 monophasic *S*. Typhimurium isolated from cases of salmonellosis in horses and cattle that occurred in Hokkaido, Japan, between 2018 and 2023; we found that all of them belonged to ST34 and were multidrug-resistant [23]. In the present study, 36.8% (7/19) of the monophasic *S*. Typhimurium isolated from food workers were classified as ST19. As monophasic *S*. Typhimurium strains belonging to ST19 were not detected in beef or pork products in the present study, it is reasonable to assume that the primary sources of infection are food products other than these meat products. In the present study, the monophasic *S*. Typhimurium belonging to ST19 was isolated from a quail eggshell sample. In Japan, quail eggs, like chicken eggs, are a popular type of table egg and are sometimes consumed raw. Antimicrobial agents can be administered to young chickens and quails before the onset of egg-laying. However, during the laying period, the administration of antibiotics to laying hens and quails is restricted due to the potential for drug residues in eggs. If antimicrobial agents are administered to laying birds during the egg production period, the eggs produced during the withdrawal period—defined as the time required for drug residues to fall below established safety limits—must not be distributed for human consumption. Antimicrobial resistance rates of *Salmonella* isolates from laying hens are lower than those of isolates from cattle, pigs, and broilers [5,6,7,8,9,17]. Similarly, *Salmonella* isolates from quails exhibit low antimicrobial resistance rates. Investigating whether the monophasic *S*. Typhimurium belonging to ST19 is maintained in quail farms in Japan and characterizing their antimicrobial resistance profiles could contribute to elucidating the transmission routes to humans of the monophasic *S*. Typhimurium belonging to ST19.

In this study, food samples were primarily collected from retail stores located near the authors’ research facilities, and *Salmonella* isolates from the feces of food workers were obtained from employees of a single fast-food company. Therefore, the findings may not be representative of the entire eastern region of Japan. Additionally, the study focused exclusively on livestock products (pork, beef, and quail eggs) and did not include other potential sources of *Salmonella*, such as fresh produce (e.g., vegetables and fruits) or seafood. The limited geographic coverage, the relatively small sample size, and the restricted range of food types examined may have restricted the comprehensiveness of the findings. Moreover, the study did not include seasonal data, and there is no direct evidence linking isolates from food to human infections. To improve future investigations, it would be valuable to (i) expand sample collection to multiple geographic regions and diverse retail outlets, (ii) include a wider variety of food categories, particularly fresh produce and seafood, (iii) collaborate with additional food service companies to obtain a broader range of *Salmonella* isolates from food workers, and (iv) employ whole-genome sequencing followed by single-nucleotide polymorphism analysis and core genome MLST to provide higher resolution for tracing transmission pathways and identifying infection sources with greater accuracy.

## 5. Conclusions

Under the Food Sanitation Act in Japan, restaurants are prohibited from serving raw beef and pork dishes, including liver, and retail stores are required to label raw beef and pork, including liver, as “for cooking (must be heated before consumption)” when sold. The background of this regulation lies in outbreaks of foodborne illness, including fatal cases of enterohemorrhagic *Escherichia coli* infection caused by the consumption of raw beef [24] and fatal cases of hepatitis E associated with the consumption of pork [25,26]. Although the law does not specify detailed heating conditions, these products must be at least superficially cooked, lightly boiled, or otherwise heat-treated before consumption. Contrastingly, no such restrictions apply to poultry or quail meat and eggs, allowing these products to be served or consumed raw or undercooked. This regulatory difference likely contributes to the higher incidence of *Salmonella*-related foodborne illnesses associated with chicken, quail, and eggs in Japan. To mitigate *Salmonella* infections, it is important to continue monitoring the prevalence and characteristics of *Salmonella* in both food products and humans. Disseminating these findings and raising awareness of the potential risks associated with each food type may help reduce infection. Our findings suggest that thorough cooking of chicken and pork meat, as well as liver, is critical to minimizing the risk of *Salmonella* transmission.

## Figures and Tables

**Table 1:** *Salmonella* prevalence in livestock products.

Animal	Sample	Sampling Period	No. of Samples	No. of Positive Samples (%)
Quail	Egg content (domestic)	March 2022 to March 2023	60	0 (0.0)
	Egg shell (domestic)	March 2022 to March 2023	60	1 (1.7)
Pig	Minced pork (domestic)	December 2022 to December 2023	40	1 (2.5) *
	Liver (domestic)	June 2023 to June 2025	30	13 (43.3) *
Cattle	Minced beef (domestic)	April 2024 to December 2024	40	0 (0.0)
	Liver (domestic)	April 2024 to December 2024	62	0 (0.0)
Chicken	Thigh meat (imported)	April 2022 to June 2025	87	18 (20.7)
	Brazil		68	9 (13.2) **
	Thailand		15	9 (60.0) **
	USA		2	0 (0.0)
	Lithuania		2	0 (0.0)

*: statistically significant difference (*p* < 0.05) relative to minced meat and pork liver samples. **: statistically significant difference (*p* < 0.05) relative to Brazil and Thailand samples.

**Table 2:** Number and serovars of *Salmonella* isolates recovered from food workers and livestock products.

Serovar	Food Worker	Pork Liver	Minced Pork	Imported Chicken Thigh Meat (Country)	Quail Egg Shell
*S*. Schwarzengrund	27	0	0	1 (Brazil)	0
*S*. Manhattan	20	0	0	0	0
Monophasic *S*. Typhimurium	19	11	1	2 (Brazil and Thailand)	1
*S*. Thompson	19	0	0	0	0
*S*. Infantis	18	0	0	0	0
*S*. Braenderup	14	0	0	0	0
*S*. Senftenberg	12	0	0	0	0
*S*. Typhimurium	9	0	0	0	0
*S*. Agona	9	0	0	3 (Thailand)	0
*S*. Rissen	6	0	0	0	0
*S*. Mbandaka	6	0	0	0	0
*S*. Derby	5	2	0	0	0
*S*. Stanley	5	0	0	0	0
*S*. Weltevreden	4	0	0	0	0
*S*. Newport	3	0	0	1 (Brazil)	0
*S.* Montevideo	3	0	0	0	0
*S.* Bareilly	3	0	0	0	0
*S*. Corvallis	3	0	0	0	0
*S.* Enteritidis	3	0	0	1 (Thailand)	0
*S.* Anatum	3	0	0	0	0
*S.* Saintpaul	2	0	0	1 (Thailand)	0
*S.* Chester	2	0	0	0	0
*S.* Singapore	2	0	0	0	0
*S.* Tennessee	2	0	0	0	0
*S.* Cubana	2	0	0	0	0
*S.* Muenster	2	0	0	0	0
*S.* Kentucky	2	0	0	1 (Thailand)	0
*S.* Albany	1	0	0	1 (Thailand)	0
*S.* Minnesota	0	0	0	5 (Brazil)	0
*S.* Heidelberg	0	0	0	1 (Brazil)	0
*S.* Olso	0	0	0	1 (Thailand)	0
Others (19 serovars and 7 untypeable)	26	0	0	0	0
Total	232	13	1	18	1

**Table 3:** Antimicrobial susceptibility of *Salmonella* isolates recovered from food workers and livestock products.

Origin	No.	ABPC	CEZ	CTX	SM	GM	KM	TC	NA	CPFX	CL	CP	TMP
		No. (%)	No. (%)	No. (%)	No. (%)	No. (%)	No. (%)	No. (%)	No. (%)	No. (%)	No. (%)	No. (%)	No. (%)
Food worker	232	27 (11.6)	3 (1.3)	2 (0.9)	113 (48.7)	3 (1.3)	28 (12.1)	69 (29.7)	15 (6.5)	3 (1.3)	2 (0.9)	11 (4.7)	30 (12.9)
Pork liver	13	8 (61.5)	5 (38.5)	0 (0.0)	12 (92.3)	0 (0.0)	0 (0.0)	10 (76.9)	0 (0.0)	0 (0.0)	0 (0.0)	6 (46.2)	8 (61.5)
Minced pork	1	1 (100.0)	0 (0.0)	0 (0.0)	1 (100.0)	0 (0.0)	0 (0.0)	1 (100.0)	0 (0.0)	0 (0.0)	0 (0.0)	1 (100.0)	1 (100.0)
Imported chicken	18	12 (66.7)	6 (33.3)	6 (33.3)	9 (50.0)	2 (11.1)	3 (16.7)	11 (61.1)	10 (55.6)	1 (5.6)	1 (5.6)	0 (0.0)	4 (22.2)
Quail egg shell	1	0 (0.0)	0 (0.0)	0 (0.0)	0 (0.0)	0 (0.0)	0 (0.0)	0 (0.0)	0 (0.0)	0 (0.0)	0 (0.0)	0 (0.0)	0 (0.0)

ABPC: ampicillin, CEZ: cefazolin, CTX: cefotaxime, SM: streptomycin, GM: gentamycin, KM: kanamycin, TC: tetracycline, NA: nalidixic acid, CPFX: ciprofloxacin, CL: colistin, CP: chloramphenicol, TMP: trimethoprim.

**Table 4:** Antimicrobial resistance profiles of *Salmonella* isolates recovered from food workers and livestock products.

Serotype	Antimicrobial Resistance Profile	Food Worker (ST)	Pork Liver (ST)	Minced Pork Meat (ST)	Imported Chicken Thigh Meat (Country, ST)	Quail Egg Shell (ST)
*S*. Schwarzengrund	SM + KM + TC + NA + CPFX	1	0	0	0	0
	SM + KM + TC + NA + TMP	2	0	0	0	0
	SM + KM + TC + TMP	9	0	0	0	0
	KM + NA + TMP	1	0	0	0	0
	SM + TMP	1	0	0	0	0
	SM + TC	1	0	0	0	0
	SM	2	0	0	0	0
	KM	4	0	0	0	0
	TMP	1	0	0	0	0
	Susceptible	5	0	0	1 (Brazil)	0
*S*. Manhattan	SM + TC + CL	1	0	0	0	0
	SM + TC + NA	1	0	0	0	0
	SM + TC	15	0	0	0	0
	CL	1	0	0	0	0
	Susceptible	2	0	0	0	0
Monophasic *S*. Typhimurium	SM + TC + NA + CPFX + CP + TMP	1 (ST34)	0	0	0	0
	ABPC + CEZ + SM + TC + CP + TMP	0	4 (ST34)	0	0	0
	ABPC + SM + TC + CP + TMP	3 (ST34)	2 (ST34)	1 (ST34)	0	0
	ABPC + CEZ + SM + TC + TMP	0	1 (ST34)	0	0	0
	ABPC + SM + KM + TC	1 (ST34)	0	0	0	0
	ABPC + CEZ + SM + KM	1 (ST19)	0	0	0	0
	ABPC + SM + TMP	1 (ST34)	1 (ST34)	0	0	0
	ABPC + SM + TC	4 (ST34)	0	0	1 (Thailand, ST34)	0
	SM + TC	0	3 (ST34)	0	0	0
	ABPC + SM	2 (ST34)	0	0	1 (Brazil, ST34)	0
	TC	2 (ST19)	0	0	0	0
	SM	1 (ST19)	0	0	0	0
	CP	1 (ST19)	0	0	0	0
	Susceptible	2 (ST19)	0	0	0	1 (ST19)
*S*. Thompson	SM + TC	1	0	0	0	0
	SM	8	0	0	0	0
	TMP	1	0	0	0	0
	Susceptible	9	0	0	0	0
*S*. Infantis	SM + KM + TC + TMP	3	0	0	0	0
	SM + KM + TC	1	0	0	0	0
	SM + TC	1	0	0	0	0
	ABPC + SM	1	0	0	0	0
	SM + TMP	1	0	0	0	0
	SM	1	0	0	0	0
	Susceptible	10	0	0	0	0
*S*. Braenderup	SM	3	0	0	0	0
	Susceptible	11	0	0	0	0
*S*. Minnesota	ABPC + CEZ + CTX + KM + TC + NA + TMP	0	0	0	1 (Brazil)	0
	ABPC + CEZ + CTX + SM + TC + NA	0	0	0	1 (Brazil)	0
	ABPC + CEZ + CTX + TC + NA	0	0	0	2 (Brazil)	0
	KM + TC + NA	0	0	0	1 (Brazil)	0

ST: sequence type.

## Data Availability

Data are contained within the article. Further inquiries can be directed to the corresponding authors.

## Abbreviations

The following abbreviations are used in this manuscript:

MLST	Multilocus Sequence Typing
BPW	Buffered Peptone Water
CLSI	Clinical and Laboratory Standards Institute
ST	Sequence Type
